# Screening for cognition in amyotrophic lateral sclerosis: test characteristics of a new screen

**DOI:** 10.1007/s00415-021-10423-x

**Published:** 2021-02-06

**Authors:** Emma Beeldman, Rosanne Govaarts, Marianne de Visser, Michael A. van Es, Yolande A. L. Pijnenburg, Ben A. Schmand, Joost Raaphorst

**Affiliations:** 1 Department of Neurology, Amsterdam UMC, Academic Medical Centre, University of Amsterdam, P.O. Box 22700, Amsterdam, The Netherlands; 2 Department of Neurology, University Medical Centre Utrecht Brain Centre, Utrecht, The Netherlands; 3 Alzheimer Centre, Amsterdam UMC, Vrije Universiteit, Amsterdam, The Netherlands; 4 Department of Medical Psychology, Amsterdam UMC, University of Amsterdam, Amsterdam, The Netherlands

**Keywords:** Cognitive screening tool, Amyotrophic lateral sclerosis, Cognitive impairment, ALS-FTD-Cog, ECAS

## Abstract

Cognitive and behavioural impairment in amyotrophic lateral sclerosis (ALS) negatively influences the quality of life and survival, and, therefore, screening for these impairments is recommended. We developed a cognitive screening tool, the amyotrophic lateral sclerosis–frontotemporal dementia–cognitive screen (ALS–FTD–Cog) and aimed to validate it in patients with ALS. During the current study, the Edinburgh Cognitive and Behavioural ALS Screen (ECAS) was published and we, therefore, decided to compare these two cognitive screening methods. The ALS–FTD–Cog was administered to 72 patients with ALS, 21 patients with behavioural variant FTD (bvFTD) and 34 healthy controls. Twenty-nine patients with ALS underwent the ECAS. ROC curve analyses were performed and sensitivity and specificity of the ALS–FTD–Cog and ECAS were calculated, with a neuropsychological examination (NPE) as the gold standard. Cognitive impairment was present in 28% of patients with ALS. ROC curve analyses of the ALS–FTD–Cog and ECAS showed an area under the curve (AUC) of 0.72 (95% CI 0.58–0.86) and 0.95 (95% CI 0.86–1.03), respectively. Compared to a full NPE, sensitivity and specificity of the ALS–FTD–Cog were 65.0% and 63.5% and of the ECAS 83.3% and 91.3%, respectively. The sensitivity and specificity of the ALS–FTD–Cog in patients with bvFTD were 94.4% and 100%, respectively. Test characteristics of the ALS–FTD–Cog were moderate, suggesting restricted practical value, as compared to a comprehensive NPE. The ECAS had an excellent AUC and high sensitivity and specificity, indicating that it is a valid screening instrument for cognitive impairment in ALS.

**Supplementary Information:**

The online version contains supplementary material available at 10.1007/s00415-021-10423-x.

## Introduction

Cognitive impairment is present in 30–50% of patients with amyotrophic lateral sclerosis (ALS) and negatively influences survival and quality of life [1–4]. Investigation of cognition, in addition to behaviour, is, therefore, recommended in patients diagnosed with ALS [5, 6].

The gold standard for measuring cognitive impairment is a full neuropsychological examination (NPE), which should be adapted to avoid bias due to impaired dexterity and speech [5]. An NPE is often a time-consuming procedure which might be a burden for the patient and not readably available in every neuromuscular clinic. Therefore, a concise screening tool could be useful. Currently, there are multiple cognitive screening tools available, but at the start of our study, only the ALS-cognitive behavioural screen (ALS-CBS) and the Penn State Screening examination of Frontal and Temporal dysfunction Syndromes (PSSFTS) were published [7–11]. These screens are concise, with an administration time of 5–10 min [12]. However, not all cognitive domains known to be affected in ALS are included in these screens, i.e. tests of social cognition are lacking [13].

The aim of the current study was to investigate the clinical validity of a new cognitive screening tool, the ALS–frontotemporal dementia–cognitive screen (ALS–FTD–Cog), which aims to cover the complete cognitive profile of ALS [13]. It consists of four frequently used cognitive tests. We hypothesized that the sensitivity of the screen would be high, as these tests have previously been demonstrated to show impairment in ALS patients. Furthermore, we expected the screen to be feasible in patients with ALS and widely applicable as the individual tests of the screen are not hampered by physical or speech impairment and normative data are available [13–16].

During our study, the Edinburgh Cognitive and Behavioural ALS Screen (ECAS) was published, which has become a widely used screening tool for cognitive impairment in ALS [9]. We, therefore, compared test characteristics of the ALS–FTD–Cog and ECAS in a subset of our study sample.

## Methods

### Participants

Patients with ALS were recruited from our tertiary referral centres (Amsterdam University Medical Centers and University Medical Center Utrecht) in the Netherlands. All patients (sporadic or familial) had a diagnosis of probable or definite ALS[17], a symptom duration of less than 12 months and an upright forced vital capacity of > 70%, as described previously [18]. We also included a positive control group of (sporadic or familial) patients with behavioural variant FTD (bvFTD)[19] with or without ALS from our tertiary referral centre (Alzheimer Centre, Amsterdam University Medical Centers). A negative control group consisted of healthy controls without a history of neurological or psychiatric disease, who were approached through social media. All participants had to be older than 18 years, had to have a reliable informant and had to be fluent in Dutch.

The local medical ethical committees of the participating hospitals approved the study. Written informed consent was obtained from all participants. This study was performed in agreement with the Declaration of Helsinki.

### Procedures

#### The ALS–FTD–Cog

The ALS–FTD–Cog is a screening tool which consists of the faux pas test (FPT, social cognition), Rivermead behavioural memory test—story recall (RBMT, verbal memory), letter fluency index (LFI, executive function) and the Boston naming test (BNT, language) [13]. All tests have validated norm scores, adjusted for age and education. The RBMT, LFI and BNT have previously shown to be impaired in ALS patients [14–16]. Social cognition deficits have more recently been recognized in ALS [20, 21]. A recent meta-analysis showed comparable effect sizes for tests of the theory of mind and facial emotion recognition, suggesting that both concepts of social cognition are impaired in patients with ALS [22]. The ALS–FTD–Cog was administered during a home visit by a trained member of the research team, in a quiet room without distractions.

#### Neuropsychological examination

A full neuropsychological examination was performed in the outpatient clinic in all participants, as described previously, within 4 weeks from the administration of the screen [18]. Cognitive tests were chosen that were not hampered by motor or speech disabilities, or adaptations were made (see online supplemental material). Alternate forms of the BNT, LFI and RBMT were used in the neuropsychological examination and the ALS–FTD–Cog. Test scores were considered abnormal when below the 5th percentile, demographically corrected. Cognitive impairment was defined according to the Strong criteria [5]. Therefore, only tests of fluency, language, executive functions and social cognition were taken into consideration. Participants were considered to be cognitively impaired when they had impaired letter fluency, or impairment on at least two non-overlapping executive functions tests or two non-overlapping language tests [5].

#### ECAS

A subset of patients with ALS, diagnosed at the outpatient neurology clinic of the University Medical Center Utrecht, underwent the ECAS within three months of the administration of the ALS–FTD–Cog. The ECAS was administered by a trained member of the research team. The ECAS (13 items) consists of an ALS-specific and ALS-non-specific part. The ALS-specific part consists of tests of language, fluency and executive functions. The ALS-non-specific part consists of tests of memory and visuospatial functions. The two parts combined produce an ECAS total score. The ECAS was considered abnormal when below predefined cut-off values (ECAS total score ≤ 105 points and ECAS ALS-specific score ≤ 77 points) [9].

#### Other measures

Behavioural impairment was assessed in all participants with the ALS-FTD-Questionnaire (ALS-FTD-Q) and the Motor Neuron Disease Behaviour scale (MiND-B) [23, 24]. Disease severity and respiratory function were measured in ALS patients with the ALS functional rating scale-revised (ALSFRS-R) and forced vital capacity (FVC), respectively [25]. Affective symptoms were measured with the Hospital Anxiety and Depression Scale (HADS) in all participants [26]. For a detailed description of all measures, see online supplemental material.

### Clinimetric evaluation of the ALS–FTD–Cog

The gold standard for cognitive impairment was the neuropsychological examination. Cognitive impairment was defined as impaired letter fluency and/or impairment on at least two non-overlapping executive functions tests and/or two non-overlapping language tests, according to the Strong criteria [5].

Tentative cut-off scores of the ALS–FTD–Cog were investigated by two means in patients with ALS:

1. The ALS–FTD–Cog was considered abnormal when ≥ 1 test was below the 5th percentile, demographically corrected. The sensitivity and specificity were calculated, as compared to the neuropsychological examination.
2. A ROC curve analysis was performed for the ALS–FTD–Cog mean T score (mean of T scores of all four items). Youden’s J statistic was used to determine the optimal cut-off value.

For comparison, the sensitivity and specificity of the ECAS were calculated in a subset of patients with ALS, using cut-off scores as described above [9].

Sensitivity and specificity of the ALS–FTD–Cog were also calculated in the bvFTD and healthy control group. We expected to find a high percentage of cognitive impairment in patients with bvFTD and a low percentage in the healthy control group.

We also assessed associations of the ALS–FTD–Cog with other measures. We, therefore, calculated correlations of the ALS–FTD–Cog with measures of cognition (NPE total score, i.e. the sum of T scores of all items, the ECAS total score and the ECAS ALS-specific score), behaviour (ALS-FTD-Q and MiND-B), physical impairment (ALSFRS-R and FVC) and affective symptoms (HADS).

### Statistical analysis

The sensitivity and specificity of the ALS–FTD–Cog, ECAS total score and ECAS ALS-specific score were calculated by means of contingency tables. Furthermore, ROC curve analyses were performed of the ALS–FTD–Cog mean T score, the ECAS total score and ECAS ALS-specific score, and Youden’s J statistic was calculated. The correlations between cognitive (ALS–FTD–Cog (mean T score), NPE (total T score), ECAS total score and ECAS ALS-specific score), behavioural (ALS-FTD-Q and MiND-B) and other measures (ALSFRS-R, FVC, HADS anxiety and HADS depression) were expressed as Spearman rank correlation coefficients (*r*_s_). Multiple imputation was performed with iterative Markov chain Monte Carlo method for missing neuropsychological test results (30/1524 data points (2.0%)). Statistical significance level was set at *p* = 0.05. Analyses were performed in PASW statistics, version 26 (SPSS).

## Results

### Participants

We included 72 ALS patients, 21 bvFTD patients (of whom 5 had concurrent ALS) and 34 healthy controls (Table 1 and online supplemental material). A subset of 29 ALS patients (40.3%), age- and education matched with the healthy controls, had been administered the ECAS.

**Table 1:** Participant characteristics

	ALS		bvFTD	HC
	Total (*n* = 72)	ECAS sample (*n* = 29/72)	(*n* = 21)	(*n* = 34)
Age	62.6 (10.0)*	62.0 (8.7)	64.6 (10.0)*	58.4 (10.1)
Sex (m/f)	50/22*	20/9*	17/4*	14/20
Education (years)	14.0 (3.0)	14.2 (2.6)	14.5 (2.2)	14.9 (1.9)
Disease duration (months)	9.0 (4–16)	9.0 (5–13)	29.0 (9–166)	n/a
Site of onset (l/b/lb)	48/22/2	21/6/2	n/a	n/a
ALSFRS-R	40.0 (28–47)	40.0 (30–47)	n/a	n/a
FVC (%pred)	92.5 (15.8)	93.8 (15.3)	n/a	n/a
HADS anxiety	4.0 (0–13)*	4.0 (0–12)	5.0 (0–12)	3.0 (0–7)
HADS depression	2.0 (0–11)*	2.0 (0–10)	3.0 (0–8)*	0.5 (0–8)
*C9orf72* mutation	4^	2^	3^	n/a
Survival (mo)	25.5 (7–67)^^	27.0 (15–67)	n/a	n/a
ALS-FTD-Q	13.3 (10.3)**	9.3 (7.4)	43.8 (12.7)**	6.4 (6.8)
MiND-B^#^	34.3 (3.0)*	35.0 (1.5)*	26.4 (6.3)**	35.7 (0,8)

Data are presented as mean (SD) or median (range), when appropriate

*l* limb onset, *b* bulbar onset, *lb* both limb and bulbar onset, *ALSFRS-R* ALS functional rating scale—revised, *FVC (%pred)* forced vital capacity, percentage of predicted value, *n/a* not applicable. Statistical differences were examined between each of the patient groups and HC

*p < 0.05; **p < 0.001; ^C9orf72 mutation status was missing in 11 patients (total cohort), 2 patients (ECAS cohort) and 8 bvFTD patients. ^^To date (checked on 22 November 2020) 66 ALS patients are deceased. ^#^ALS N = 57, HC N = 26, FTD N = 19. ^##^ALS N = 21. The participants who were administered the ECAS were patients who visited the out-patient clinic of the University Medical Center Utrecht, and therefore can be considered a random (geographic) sample. The mean interval between the administration of the ALS–FTD–Cog and ECAS was 41 days (SD 25). A part of the current cohort has been published previously [18]

### Cognitive test results

Twenty ALS patients (27.8%) had cognitive impairment, based on the NPE and the Strong criteria, mostly in the social cognition (*n* = 20) and executive functions (*n* = 12) domains (online supplemental material).

Thirty-two ALS patients (44.4%) were impaired on one (*n* = 23) or more (*n* = 9) tests of the ALS–FTD–Cog (online supplemental material). The faux pas test (social cognition) was most frequently impaired (*n* = 28).

Respectively, nine and seven patients with ALS (out of 29, 31.0% and 24.1%) had an abnormal ECAS total score and ECAS ALS-specific score. Five of these patients had cognitive impairment on the neuropsychological examination.

According to the ALS–FTD–Cog, twelve patients were classified as cognitively impaired, all of whom had no cognitive impairment on the NPE. According to the ECAS total score and ECAS ALS-specific score, one and three patients, respectively, were classified as cognitively impaired, all of whom had no cognitive impairment on the NPE (Table 2).

**Table 2:** Cognitive impairment in ALS patients (*n* = 29) based on NPE, ALS–FTD–Cog and ECAS

	NPE	ALS–FTD–Cog	ECAS total	ECAS ALS specific
Cognitive impairment	6 (20.7%)	12 (41.4%)	7 (24.1%)	9 (31.0%)
No cognitive impairment	23 (79.3%)	17 (58.6%)	22 (75.9%)	20 (69.0%)

*NPE* neuropsychological examination, *ALS–FTD–Cog* amyotrophic lateral sclerosis–frontotemporal dementia–cognitive screen, *ECAS* Edinburgh Cognitive and Behavioural ALS Screen

Eighteen patients with (ALS-)bvFTD (86%) had cognitive impairment based on the neuropsychological examination, mostly in the domains social cognition (*n* = 18), executive functions (*n* = 18) and verbal memory (*n* = 15). Seventeen of these patients had an abnormal ALS–FTD–Cog. One healthy control (2.9%) had cognitive impairment based on the neuropsychological examination, in the domains social cognition and executive functions.

According to the ALS-FTD-Q, ten patients with ALS (13.9%) had mild behavioural impairment and six patients (8.3%) fulfilled the criteria for bvFTD [19]. Twelve patients (out of 57, 21.1%) had behavioural impairment according to the MIND-B [24].

### Test characteristics of the ALS–FTD–Cog

The median administration time in ALS patients was 40 min (range 28–61). The scores on the subtests of the ALS–FTD–Cog are shown in the supplemental material for all participant groups.

#### Sensitivity and specificity

When ≥ 1 impaired test of the ALS–FTD–Cog was considered abnormal, the sensitivity and specificity in ALS patients using the NPE as gold standard were 65.0% and 63.5%, respectively (online supplemental material). The sensitivity and specificity in (ALS-)bvFTD patients were 94.4% and 100%, respectively.

The ROC curve analysis of the ALS–FTD–Cog mean T score showed an AUC of 0.72 (95% CI 0.58–0.86), with a Youden’s J statistic of 0.4. The optimal cut-off value was 46.9, with a corresponding sensitivity of 65% and specificity of 75% (Table 3).

**Table 3:** ROC curve analysis and Youden’s J statistic of the ALS–FTD–Cog and ECAS

		ALS (*n* = 74)	ALS (*n* = 29)	bvFTD (*n* = 21)	HC (*n* = 34)
ALS–FTD–Cog mean T score	AUC	0.72 (0.58–0.86)	0.60 (0.30–0.90)	0.78 (0.56–0.99)	0.61 (0.44–0.77)
	Youden’s J	0.4	0.33	0.61	0.61
ECAS total	AUC	n/a	0.90 (0.78–1.01)	n/a	n/a
	Youden’s J	n/a	0.83	n/a	n/a
ECAS ALS specific	AUC	n/a	0.95 (0.86–1.03)	n/a	n/a
	Youden’s J	n/a	0.78	n/a	n/a

*ALS–FTD–Cog* amyotrophic lateral sclerosis–frontotemporal dementia–cognitive screen, *ECAS* Edinburgh Cognitive and Behavioural ALS Screen, *ALS* amyotrophic lateral sclerosis, *bvFTD* behavioural variant frontotemporal dementia, *HC* healthy controls, *AUC* area under the curve

Youden’s J statistic is calculated with the formula sensitivity + specificity−1

The sensitivity of both the ECAS total score and ECAS ALS-specific score was 83.3% in ALS patients, using the NPE as the gold standard. The specificity of the ECAS total and ALS-specific score in ALS patients was 82.6% and 91.3%, respectively (online supplemental material). The ROC curve analyses of the ECAS total score and ECAS ALS-specific score showed an AUC of 0.90 (95% CI 0.78–1.01) and 0.95 (95% CI 0.86–1.03), respectively, with a Youden’s J statistic of 0.83 and 0.78, respectively (Table 3).

#### Associations of ALS–FTD–Cog with measures of cognition, behaviour, physical impairment and affective symptoms

The correlation of the ALS–FTD–Cog scores with the NPE was moderate (*r*_s_ 0.54, p < 0.001) and weak with the ECAS total score and ECAS ALS-specific score (*r*_s_ 0.34, *p* = 0.08 and *r*_s_ 0.25, *p* = 0.2, respectively. The correlation of the NPE with the ECAS total score and ALS-specific score was moderate (*r*_s_ 0.51 and *r*_s_ 0.49, respectively, *p* < 0.01). Correlations of the ALS–FTD–Cog scores with the ALS-FTD-Q, ALSFRS-R, FVC, HADS anxiety and HADS depression were weak (online supplementary material).

## Discussion

We investigated the clinical validity of a new cognitive screening tool, the ALS–FTD–Cog in a cohort of ALS patients with a short disease duration (symptom onset < 12 months) with a prevalence of cognitive impairment of nearly 30%, which is comparable to large population-based cohort studies [27, 28]. The sensitivity and specificity of the ALS–FTD–Cog in ALS patients were moderate and do not justify its use in clinical practice. The sensitivity and specificity of the ALS–FTD–Cog in bvFTD patients were high, indicating that the screen detects cognitive impairment as seen in bvFTD. In a subset of 29 patients, a high sensitivity and specificity of another, widely used cognitive screening instrument, the ECAS, was found.

### Screening for cognitive impairment

A screening test should be easy to administer and score, widely applicable, time efficient with a high sensitivity to select patients who may need further testing, and a high specificity to preclude unnecessary further testing. The ALS–FTD–Cog is easy to administer although basic training in administering and scoring cognitive tests is needed. The ALS–FTD–Cog is widely applicable as it is composed of internationally validated tests with available normative data. The administration time is quite long (40 min) which is similar to the ECAS [12].

However, the moderate sensitivity of the ALS–FTD–Cog (65%) indicates that it is less suitable as a screening tool. This could be caused by multiple factors. First, we might have included the wrong tests in the screen. The faux pas test, a measure of theory of mind, proved difficult to interpret for both the participant and the administrator. In our study, most participants, including healthy controls, had problems attributing only one emotion to the situation at hand. This led to a high number of abnormal empathy scores. When we would have excluded the empathy score from the screen and only consider the faux pas total score, this would have resulted in a decrease in the sensitivity. A recent study of social cognition in bvFTD and other neurodegenerative and psychiatric disorders found that the faux pas test does not differentiate bvFTD patients from the other participant groups. However, the Ekman 60 faces test, which was included in our NPE, but not in the screen, showed a high discriminating rate in a previous study [29]. The discriminating rate of the Ekman 60 faces test was also shown in a meta-analysis, comparing patients with bvFTD to patients with Alzheimer’s disease and healthy controls [30]. In ALS, especially the recognition of disgust and surprise seems impaired [22].

Another test that may have caused a limited sensitivity of our screen is the Rivermead behavioural memory test (RBMT). The current consensus criteria for cognitive impairment in ALS do not include memory impairment [5]. Our selection of tests was based on our meta-analysis of the cognitive profile of ALS that showed a large effect size for verbal memory impairment and evidence from multiple imaging and pathological studies showing hippocampal involvement in ALS [13, 31–34]. In the current study, the RBMT was abnormal in 8 patients (11.1%), of whom 7 also had abnormal tests in the executive domain, which reflects the low prevalence of isolated memory impairment in ALS [28]. Thus, the RBMT had limited added value for the detection of cognitive impairment in ALS, although it increases the internal consistency of the screen.

Second, the moderate clinimetric properties of the ALS–FTD–Cog might be related to the inclusion of a limited number of tests. We included four complete neuropsychological tests in the ALS–FTD–Cog, instead of a higher number of separate items of (sub)tests, hypothesizing that the availability of demographically corrected normative data would result in feasibility (no need to generate new normative scores) and a high sensitivity. In comparison, the ECAS includes separate items of 13 neuropsychological tests [9]. This approach leads to the investigation of different facets of multiple cognitive domains, of which the potential benefit, i.e. a higher sensitivity, has been suggested previously [12]. However, the scoring of such a screening tool is not based on established normative data and the weight of the scores of the different tests is seemingly random. The reported sensitivity and specificity of the ECAS range between 50 and 100% and 80 and 95%, respectively [12, 35–37]. In our small study population, the previously reported high sensitivity of the ECAS was confirmed when using the original cut-off values. In combination with previous studies which have shown good clinimetric properties of the ECAS, our results indicate that the ECAS is a valid screening instrument for cognitive impairment in ALS.

### Future directions of the ALS–FTD–Cog

Even though we do not recommend the use of the ALS–FTD–Cog in ALS patients, we did find excellent clinimetric properties of the screen in our group of bvFTD patients. However, cognitive screens are most informative at the moment of diagnosis, whereas our bvFTD patients had a more advanced disease stage, reflected by a disease duration ranging from 9 to 166 months and severe cognitive impairment based on the NPE. We recommend examining the clinimetric properties of the ALS–FTD–Cog in a group of newly diagnosed bvFTD patients.

### Limitations

The ALS–FTD–Cog was developed before the publication of the Strong criteria for cognitive impairment in ALS [5]. The screen includes only one test per cognitive domain, and, therefore, it is not possible to fulfil the Strong criteria (which require abnormal scores on two executive or language tests), except for verbal fluency impairment.

Less than half of the ALS patients were administered the ECAS (40.3%), because it was not yet published at the beginning of our study. Also, a small minority of participants had missing data on the neuropsychological examination for which multiple imputation was used.

## Conclusion

The ALS–FTD–Cog had moderate sensitivity and specificity in our cohort of patients with ALS when compared to the gold standard and we do not recommend its clinical use in patients with ALS, although the clinimetric properties in bvFTD patients are excellent. Regarding the ECAS, we were able to corroborate a previously reported high sensitivity and specificity in a small subset of patients, indicating that it is a valid screening tool for cognitive impairment in ALS.

## Supplementary Information

Below is the link to the electronic supplementary material.

Supplementary file1 (DOCX 65 KB)

## Data Availability

Data are available upon request.
