# Supplementary material for: Screening for cognition in amyotrophic lateral sclerosis: test characteristics of a new screen
Source: J Neurol. 2021 Feb 6;268(7):2533–40. doi: 10.1007/s00415-021-10423-x (PMC8217007; doi:10.1007/s00415-021-10423-x)
Supplement: Supplementary file 1 — Supplementary file1 (DOCX 65 KB) [file 415_2021_10423_MOESM1_ESM.docx]

**Supplemental material**

**Page**

1. Neuropsychological examination 2
2. Other measures 3
3. Participant selection and inclusion 4
4. Scores on the ALS-FTD-Cog 5
5. ROC curve analysis of the ALS-FTD-Cog and ECAS in 29 patients with ALS 6
6. Contingency tables of the ALS-FTD-Cog and ECAS, compared to the neuropsychological 7
   examination
7. Associations of the ALS-FTD-Cog with measures of cognition, behaviour, physical impairment 8
   and affective symptoms
8. Percentage of ALS patients impaired on subtests of the ECAS and ALS-FTD-Cog 9
9. References 10
10. **Neuropsychological examination

    Table 1. Neuropsychological examination**

| **Order** | **Cognitive test** | **Cognitive domain** | **Items (N)** | **Parallel version** |
| --- | --- | --- | --- | --- |
| 1 | Dutch adult reading test (DART) | Verbal IQ | 50 | No |
| 2 | Benton temporal orientation test (BTOT) | Global cognition | 5 | No |
| 3 | Anti-saccade test | Executive functions | 20 | No |
| 4 | Similarities (subtest of WAIS-IV) | Language | 19 | No |
| 5 | Boston naming test (BNT) | Language | 20 | Yes |
| 6 | Judgment of line orientation (JOLO) | Visuospatial functions | 30 | No |
| 7 | Letter fluency index | Executive functions | n/a | Yes |
| 8 | Category fluency (animals, occupation, supermarket) | Executive functions | n/a | No |
| 9 | Visual association test | Visual memory | 12 or 24 | No |
| 10 | Rey auditory verbal learning test (RAVLT) | Verbal memory | 7 | Yes |
| 11 | Letter Number sequencing | Attention | 10 | No |
| 12 | Rivermead behavioral memory test (RBMT) | Verbal memory | 2 | Yes |
| 13 | Ekman 60 faces test | Social cognition | 60 | No |
| 14 | Wisconsin card sorting test (WCST) | Executive functions | n/a | No |

Legend. The neuropsychological test protocol was always performed in this order. Breaks were given when needed. For the determination of cognitive impairment, the following tests were used: anti-saccade test, similarities, Boston naming test, letter fluency index, category fluency, letter number sequencing, Ekman 60 faces test and the Wisconsin card sorting test.

1. **Other measures**

For all participants the proxy filled out the ALS-FTD-Questionnaire (ALS-FTD-Q) and the Motor neuron disease behaviour scale (MiND-B), two disease specific and validated questionnaires for behavioural changes in ALS.[1,2] A score between 22 and 28 on the ALS-FTD-Q (maximum score 100) indicates mild behavioural impairment, a score of ≥29 indicates severe behavioural impairment, consistent with bvFTD. A score below 34 on the MiND-B (maximum score 36) indicates behavioural impairment (without a distinction of mild and severe impairment). Disease severity was measured in ALS patients with the ALS functional rating scale – revised (ALSFRS-R, score ranges from 0 to 48; a higher score indicates less impairment).[3] Respiratory function was assessed with the upright forced vital capacity (FVC) in ALS patients. Symptoms of anxiety and depression were measured with the Hospital Anxiety and Depression Scale (HADS) in all participants. One item (‘I feel slowed down’) was excluded, as described previously (score ranges from 0 to 39; a higher score indicates more anxiety/depression).[4,1]

1. **Participant selection and inclusion**

**Figure 1. Flowchart of participant selection**

Invited to participate
ALS: 98
bvFTD: 41
HC: - *

Included in study
ALS: 72
bvFTD: 21
HC: 34

Did not participate**
ALS: 26
bvFTD: 18
HC: - *

ECAS administered
ALS: 29
bvFTD: 0
HC: 0

**Legend.** ALS: amyotrophic lateral sclerosis patients; bvFTD: behavioural variant frontotemporal dementia patients. This group also included 5 ALS-bvFTD patients; HC: healthy controls; ECAS: Edinburgh cognitive and behavioural ALS screen. *Healthy controls were approached through social media platforms. **Reasons to decline participation were fatigue (n=11), lack of interest (n=10) and fast progression of motor impairment (n=5).

1. **Scores on the ALS-FTD-Cog**

**Table 2. Demographically corrected scores on the ALS-FTD-Cog per participant category**

|  | **ALS (N=72)** | **bvFTD (N=21)** | **HC (N=34)** |
| --- | --- | --- | --- |
| **Faux pas test**  Total score  Empathy score | 48.5 (15.3)  34.2 (15.5)* | 26.0 (20.8)**  12.3 (12.5)** | 46.5 (8.4)  41.6 (13.2) |
| **RBMT**  Immediate  Delayed - corrected | 46.5 (8.4)  47.2 (11.2) | 28.1 (11.6)**  26.3 (26.8)** | 49.0 (9.8)  49.2 (9.3) |
| **LFI** | 43.7 (6.4)* | 14.9 (60.7)** | 40.4 (3.2) |
| **BNT** | 48.6 (9.5) | 35.8 (12.8)** | 49.6 (7.8) |

Legend. Scores of the neuropsychological tests are T-scores, shown as mean (SD). RBMT: Rivermead behavioural memory test – story recall; immediate: immediate recall; delayed – corrected: delayed recall, corrected for immediate recall score; LFI: letter fluency index; BNT: Boston naming test. Differences were examined between ALS and HC, bvFTD and HC and ALS and bvFTD. *p<0.05; **p<0.001. All test scores differed significantly between ALS and bvFTD and bvFTD and HC.

1. **ROC curve analysis of the ALS-FTD-Cog and ECAS in 29 patients with ALS**


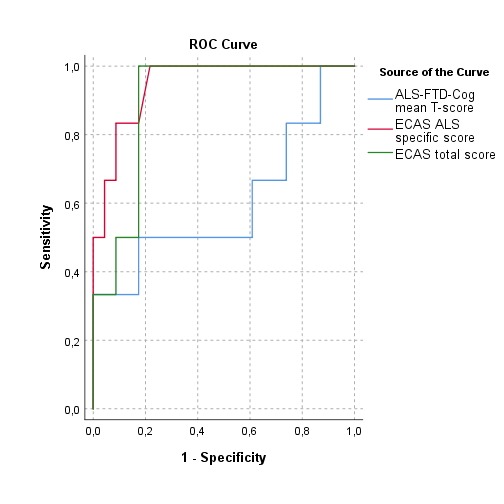


1. **Contingency tables of the ALS-FTD-Cog and ECAS, compared to the neuropsychological examination**

**Table 3a. Contingency table of ALS-FTD-Cog in ALS patients, compared to the neuropsychological examination**

|  | **NPE +** | **NPE –** | **N** |
| --- | --- | --- | --- |
| ALS-FTD-Cog + | 13 | 19 | 32 |
| ALS-FTD-Cog – | 7 | 33 | 40 |
| N | 20 | 52 | 72 |

Legend. ALS-FTD-Cog: ALS – FTD cognitive screen; NPE: neuropsychological examination; –: normal test results; +: abnormal test results. When only the total score of the faux pas test (instead of the empathy score) was taken into consideration, the number of patients with a normal ALS-FTD-Cog increased to 55, with a sensitivity and specificity of 50% and 86.5%, respectively, compared to the neuropsychological examination. Youden’s J statistic = 0.29.

**Table 3b. Contingency table of ECAS total score in ALS patients, compared to the neuropsychological examination**

|  | **NPE +** | **NPE –** | **N** |
| --- | --- | --- | --- |
| ECAS total + | 5 | 4 | 9 |
| ECAS total – | 1 | 19 | 20 |
| N | 6 | 23 | 29 |

Legend. ECAS: Edinburgh cognitive and behavioural ALS screen; NPE: neuropsychological examination; –: normal test results; +: abnormal test results.

Youden’s J statistic = 0.66.

**Table 3c. Contingency table of ECAS ALS specific score in ALS patients, compared to the neuropsychological examination**

|  | **NPE +** | **NPE –** | **N** |
| --- | --- | --- | --- |
| ECAS ALS specific + | 5 | 2 | 7 |
| ECAS ALS specific – | 1 | 21 | 22 |
| N | 6 | 23 | 29 |

Legend. ECAS: Edinburgh cognitive and behavioural ALS screen; NPE: neuropsychological examination; –: normal test results; +: abnormal test results. Youden’s J statistic = 0.75.

1. **Associations of the ALS-FTD-Cog with measures of cognition, behaviour, physical impairment and affective symptoms**

**Table 4. Spearman rank correlation coefficients of all cognitive measures in ALS patients**

|  | **ALS-FTD-Cog** | **ECAS**  **Total** | **ECAS**  **ALS specific** | **ECAS**  **not specific** |
| --- | --- | --- | --- | --- |
| NPE | 0.55** | 0.51** | 0.49** | 0.49** |
| ALS-FTD-Cog | - | 0.34 | 0.25 | 0.46* |

Legend. Correlations are expressed as Spearman rank correlation coefficients (*r_s_*). NPE: neuropsychological examination (sum of T-scores); ALS-FTD-Cog: ALS-FTD cognitive screen (mean T-score); ECAS: Edinburgh cognitive and behavioural ALS screen. **p*< 0.05, ***p*< 0.01

**Table 5. Spearman rank correlation coefficients of ALS-FTD-Cog with other measures in ALS patients**

|  | **ALS-FTD-Q** | **MiND-B** | **ALSFRS-R** | **FVC** | **HADS**  **anxiety** | **HADS depression** |
| --- | --- | --- | --- | --- | --- | --- |
| ALS-FTD-Cog | -0.32* | 0.18 | 0.02 | 0.11 | -0.15 | -0.19 |

Legend. Correlations are expressed as Spearman rank correlation coefficients (*r_s_*). ALS-FTD-Cog: ALS-FTD cognitive screen (mean T-score); ALS-FTD-Q: amyotrophic lateral sclerosis – frontotemporal dementia – questionnaire; MiND-B: Motor neuron disease – behavioural questionnaire; ALSFRS-R: ALS functional rating scale – revised; FVC: forced vital capacity; HADS: hospital anxiety and depression scale. * *p*< 0.01

1. **Percentage of ALS patients impaired on subtests of the ECAS and ALS-FTD-Cog**

**Table 6. ALS patients (n=29) impaired on subtests of the ECAS and ALS-FTD-Cog**

| **Cognitive domain** | **Impaired on ECAS (%)** | **Impaired on ALS-FTD-Cog (%)** |
| --- | --- | --- |
| Language | 14 (48.3%) | 1 (3.4%) |
| Fluency | 2 (6.9%) | 1 (3.4%) |
| Executive functions | 9 (31.0%) | 11 (37.9%)* |
| Memory | 4 (13.8%) | 5 (17.2%) |
| Visuospatial | 4 (13.8%) | n/a |
| ALS specific score | 7 (24.1%) | n/a |
| Total score | 9 (31.0%) | n/a |

Legend. ECAS: Edinburgh cognitive and behavioural ALS screen; ALS-FTD-Cog: ALS-FTD-cognitive screen. In the domain fluency the verbal fluency index was used to avoid bias due to speech disturbances and validated Dutch normative data were used to determine impairment.[5] *The faux pas test is a measure of social cognition, which is a part of the executive functions domain in the ECAS.**References**

1. Raaphorst J, Beeldman E, Schmand B, Berkhout J, Linssen WH, van den Berg LH, Pijnenburg YA, Grupstra HF, Weikamp JG, Schelhaas HJ, Papma JM, van Swieten JC, de Visser M, de Haan RJ (2012) The ALS-FTD-Q: a new screening tool for behavioral disturbances in ALS. Neurology 79: (13):1377-1383

2. Mioshi E, Hsieh S, Caga J, Ramsey E, Chen K, Lillo P, Simon N, Vucic S, Hornberger M, Hodges JR, Kiernan MC (2014) A novel tool to detect behavioural symptoms in ALS. Amyotroph Lateral Scler Frontotemporal Degener 15 (3-4):298-304. doi:10.3109/21678421.2014.896927

3. Cedarbaum JM, Stambler N, Malta E, Fuller C, Hilt D, Thurmond B, Nakanishi A (1999) The ALSFRS-R: a revised ALS functional rating scale that incorporates assessments of respiratory function. BDNF ALS Study Group (Phase III). J Neurol Sci 169 (1-2):13-21

4. Spinhoven P, Ormel J, Sloekers PP, Kempen GI, Speckens AE, Van Hemert AM (1997) A validation study of the Hospital Anxiety and Depression Scale (HADS) in different groups of Dutch subjects. Psychol Med 27 (2):363-370

5. Beeldman E, Jaeger B, Raaphorst J, Seelen M, Veldink J, van den Berg L, de Visser M, Schmand B (2014) The verbal fluency index: Dutch normative data for cognitive testing in ALS. Amyotroph Lateral Scler Frontotemporal Degener 15 (5-6):388-391. doi:10.3109/21678421.2014.906620
